# The dentate gyrus in depression: directions for future research

**DOI:** 10.1038/s41380-020-0678-8

**Published:** 2020-02-17

**Authors:** Jasper O. Nuninga, René C. W. Mandl, Iris E. C. Sommer

**Affiliations:** 1 Department of Biomedical Sciences of Cells and Systems, University Groningen, University Medical Center Groningen, Groningen, The Netherlands; 2 Department of Psychiatry, UMC Utrecht Brain Center, University Medical Center Utrecht, Utrecht, The Netherlands

**Keywords:** Neuroscience, Depression

## To the Editor:

Koch et al. [1] discuss our results regarding volume increases in the dentate gyrus (DG) [2] in the interesting context of research into stress-related disorders and fear generalization in combination with neurogenesis. While our research included severely depressed patients, Koch et al. [1] raise the possibility that findings may be generalized to wider diagnostic groups, including trauma patients (i.e., PTSD) and patients with an anxiety disorder. In the following we would like to discuss this wider interpretation of our results and shed light on the further steps needed to be taken in years to come. We recognize that our work serves as a next step into understanding the molecular mechanisms behind severe depression (and perhaps other stress-related disorders) and the development of new therapies aiming to correct pathophysiological mechanisms.

In short, using 7-tesla magnetic resonance imaging (MRI) we found that after ten sessions of electroconvulsive therapy (ECT) the volume of the DG was significantly increased in severely depressed patients, leaving the other subfields of the hippocampus unaffected. These findings point in the direction of increased neurogenesis after ECT, although other functional recovery processes (such as synaptogenesis, axonal sprouting, and angiogenesis) may also contribute to the increase in volume. In healthy controls (*n* = 8), this increase was not present. In addition, we found that baseline DG volume could predict clinical response (measured with the 17-item Hamilton Depression Rating scale; HAM-D, where higher scores indicate more severely depressed patients) in a regression model. Furthermore, we found that the change in volume was associated to a change in HAM-D score (i.e., larger treatment responses were associated to greater increases in volume). Importantly, our technical equipment has two major advantages:

1. We used ultra-high field MRI (7 tesla), which enabled us to focus on the hippocampal area with resolution of 0.286 × 0.286 × 2 mm^3^, a considerable higher image resolution than previous work.
2. We used automatic scan planning, which enabled us to rescan the exact same location using the exact same angulation on both occasions, which substantially increases sensitivity to volume changes.

As we included severely ill patients (mean baseline Hamilton score of 22.59) it was extremely challenging to motivate and engage participants to complete both scan sessions, which resulted in a small sample size (*N* = 23 patients). Our findings trigger other questions and together with Koch and other authors in the field, we can now design a path to further answer remaining questions in order to come to rapid, new, and better tolerated treatment options for severely ill patients.

In answer to the first point raised by Koch et al. [1], regarding the difference of DG baseline volume between patients and controls, we did not find a significant difference of baseline DG volumes (left and right) between patients and controls (difference left DG patients − controls = −33.03 mm^3^, Cohen’s *d* = −0.32, difference test *t* = −0.69, *p* = 0.51; difference right DG patients − controls = −19.90 mm^3^, Cohen’s *d* = −0.26, difference test *t* = −0.5, *p* = 0.63). The volumes at post treatment of the left and right DG of patients are not statistically different from that of the controls (difference left DG patients − controls = −1.38 mm^3^, Cohen’s *d* = −0.012, difference test *t* = −0.03, *p* = 0.97; difference right DG patients − controls = −0.45 mm^3^, Cohen’s *d* = −0.004, difference test *t* = −0.01, *p* = 0.99). A second question raised by Koch et al. [1], concerns the association between DG volumes and depression severity at baseline. Koch et al. state that baseline volumes of the DG could be associated to depression severity and that this association could explain the predictive effect of DG volumes. However, we do not find an association between left or right DG and depression severity at baseline in the patient group (left *r* = −0.21, *p* = 0.37, right r = 0.28 *p* = 0.24). Moreover, including depression severity at baseline in our regression model predicting clinical change, did not change our results: baseline DG volume (left/right) still predict treatment response (in the patient group). Therefore, predicting treatment response based on baseline DG volumes cannot be explained by depression severity at baseline in our sample (although the effect of depression severity could be missed due to possible type II errors). Further, left and right DG have opposite effects in the linear regression model predicting clinical response. At baseline a smaller left DG is associated with better response, while for the right DG the inverse seems true. Interestingly, when computing the widely used asymmetry index ((left − right)/(left + right) [3, 4]) for the DG, the index is able to predict the response of ECT (*t* = −3.44, *p* = 0.004). This prediction model, with age and gender as covariates, is significant and explains 45.8% of the variance (*F*(3,15) = 4.23, *p* = 0.02). Again, inclusion of baseline Hamilton scores does not significantly change the results (the asymmetry index remains a significant predictor: *t* = −2.68, *p* = 0.018). Whether this observation will hold in larger samples, and especially at conventional (3 tesla) MRI, will be a valuable clinical question. Specifically, when this observation holds at 3 tesla MRI it could be more easily implemented in the clinic to help predict clinical response for individual patients (in combination with the help of other predictors, e.g., DG-related tasks such as pattern separation).

For further research, a first important question is to answer whether or not the findings (i.e., a significant increase in volume of the DG after ECT in severely depressed patients) from our previous study [2] using ultra-high field MRI can be replicated with standard clinical MRI scanners operating at 3 tesla. To answer this first question, we collaborated with a group from Tokyo University. Together with Takamiya et al. we reanalyzed their previously published data [5], now reporting a significant correlation between changes in DG volume and clinical response after bilateral ECT in an independent sample using 3 tesla MRI [6]. In the aforementioned previously published study [5], Takamiya et al. reported volume changes in DG volumes but did not find a simple linear correlation between difference scores in DG volume and HAM-D. However, when they implemented the same repeated measures correlation [7] we used in our study [2], a significant correlation was found between a decrease in HAM-D score and an increase in right DG volume (yet not significant in left DG [6]). This finding again highlights the importance of the DG and neuroplastic changes in the DG in response to ECT treatment and suggests feasibility of replicating our findings using conventional 3 tesla MRI.

The next questions to answer regards the generalizability of our findings as well as a confirmation that they are related to neurogenesis. Now that we have a replicable method to assess DG volume and changes in that volume during recovery, we and others can set out to assess whether volumetric changes are related to plastic changes of the DG during remission. Second, animal research using both MRI and post-mortem quantification of neurogenesis is needed to confirm our theory that DG changes are caused by neurogenesis. If volumetric changes reflect plastic changes of the DG during recovery and if decreased plasticity of the DG can be confirmed to underlie the broader category of stress-related disorders, this would be a major aid to develop new treatments targeting this mechanism.

In terms of treatment, while ECT is highly effective, its tolerability is low, which restricts it use. Previous animal research has delivered a wealth of information regarding processes that can positively impact neurogenesis, which include: fasting for at least 24 h [8], physical exercise (especially running) [9], sleep [10], and demanding cognitive tasks [11]. We envision a treatment with intensive use of these four elements as an effective and noninvasive new treatment for depression and perhaps other stress-related disorders. An extra challenge will be the motivation of patients for such a combined intervention. To this end, we may use knowledge from the gaming industry to develop an attractive and engaging program that motivates even apathic participants to continue their practice in order to stimulate neurogenesis and help patients overcome their stress-related disorders. We have work to do!
