# Correction: The dentate gyrus in depression: directions for future research

**DOI:** 10.1038/s41380-020-0712-x

**Published:** 2020-04-22

**Authors:** Jasper O. Nuninga, René C. W. Mandl, Iris E. C. Sommer

**Affiliations:** 1 Department of Biomedical Sciences of Cells and Systems, University Medical Center Groningen, University Groningen, Groningen, The Netherlands; 2 Department of Psychiatry, UMC Utrecht Brain Center, University Medical Center Utrecht, Utrecht, The Netherlands

**Keywords:** Neuroscience, Depression

**Correction to: Molecular Psychiatry**

10.1038/s41380-020-0678-8  published online 17 February 2020

This article was originally published under Nature Research’s License to Publish, but has now been made available under a [CC BY 4.0] license. The PDF and HTML versions of the article have been modified accordingly.

